# Kidney Tertiary Lymphoid Structures in Lupus Nephritis Develop into Large Interconnected Networks and Resemble Lymph Nodes in Gene Signature

**DOI:** 10.1016/j.ajpath.2020.07.015

**Published:** 2020-08-17

**Authors:** Seyed Esmaeil Dorraji, Premasany Kanapathippillai, Aud-Malin Karlsson Hovd, Mikael Ryan Stenersrød, Kjersti Daae Horvei, Anita Ursvik, Stine Linn Figenschau, Dhivya Thiyagarajan, Christopher Graham Fenton, Hege Lynum Pedersen, Kristin Andreassen Fenton

**Affiliations:** ∗ RNA and Molecular Pathology Research Group, Department of Medical Biology, Faculty of Health Science, UiT Arctic University of Norway, Tromsø, Norway; † Genomic Support Center, Department of Clinical Biology, Faculty of Health Science, UiT Arctic University of Norway, Tromso, Norway

## Abstract

Immune aggregates organized as tertiary lymphoid structures (TLS) are observed within the kidneys of patients with systemic lupus erythematosus and lupus nephritis (LN). Renal TLS was characterized in lupus-prone New Zealand black × New Zealand white F1 mice analyzing cell composition and vessel formation. RNA sequencing was performed on transcriptomes isolated from lymph nodes, macrodissected TLS from kidneys, and total kidneys of mice at different disease stages by using a personal genome machine and RNA sequencing. Formation of TLS was found in anti–double-stranded DNA antibody–positive mice, and the structures were organized as interconnected large networks with distinct T/B cell zones with adjacent dendritic cells, macrophages, plasma cells, high endothelial venules, supporting follicular dendritic cells network, and functional germinal centers. Comparison of gene profiles of whole kidney, renal TLS, and lymph nodes revealed a similar gene signature of TLS and lymph nodes. The up-regulated genes within the kidneys of lupus-prone mice during LN development reflected TLS formation, whereas the down-regulated genes were involved in metabolic processes of the kidney cells. A comparison with human LN gene expression revealed similar up-regulated genes as observed during the development of murine LN and TLS. In conclusion, kidney TLS have a similar cell composition, structure, and gene signature as lymph nodes and therefore may function as a kidney-specific type of lymph node.

Systemic lupus erythematosus (SLE) and lupus nephritis (LN) are chronic autoimmune diseases characterized by inflammation and damage in the kidneys.^1^^,^^2^ The deposition of immune complexes within the glomeruli and within the tubular interstitial membranes stimulates glomerular and tubular cells to produce chemokines that attract immune cells and can also activate intrinsic immune cells, such as macrophages and dendritic cells (DCs).^3^, ^4^, ^5^, ^6^, ^7^ This deposition led to an increased accumulation of immune cells within the kidneys during the development of LN.^8^

Chronic inflammatory processes, such as infection and autoimmunity, cause tertiary lymphoid structures (TLS) to develop within different organs.^9^ These structures, resembling secondary lymphoid organs,^10^ have been detected in patients with different diseases that affect the kidneys, such as IgA nephropathies^11^ and LN,^12^^,^^13^ and in calcineurin Aα heterozygous mice,^14^ lupus-prone mice,^15^ and during aging.^16^^,^^17^ The size and location of kidney biopsies make it difficult to assess the extent of the developed TLS in human LN. Whether TLS are sites for activation or inhibition of immune cells is still not known.^15^

Changes in gene expression during the development of LN have been extensively studied in human and murine LN.^18^, ^19^, ^20^ Most of the differences in gene expression observed in diseased LN kidneys compared with healthy kidneys are involved in the activation of the innate and the adaptive immune systems.^21^ This finding indicates that the formation of TLS might be an important feature of LN. In a longitudinal study on lupus-prone, New Zealand black × New Zealand white (NZB/W) F1 mice, the formation of renal immune aggregates resembling TLS during the progression of LN has been observed.^22^ We hypothesize that the gene profile of kidney-specific TLS is similar to the lymph nodes of lupus-prone mice in an active stage of disease. The present study was undertaken to characterize the cell composition and analyze the gene expression profile of macrodissected kidney-specific TLS compared with whole kidney and lymph nodes of lupus-prone mice during the development of anti–double-stranded DNA (dsDNA) antibody production and progression of LN in three different stages of the disease.

## Materials and Methods

### Animals

NZB/W mice were obtained from Jackson Laboratory (Bar Harbor, ME). Treatment and care of animals were conducted in accordance with guidelines of the Norwegian Ethical and Welfare Board for Animal Research, and the institutional review board approved the study.

### Isolation of Lymph Nodes and Kidneys from NZB/W Mice

Seven-week–old NZB/W mice (Table1) (*n* = 5), 20- to 41-week–old mice 4 to 14 weeks anti-dsDNA–positive (*n* = 12), and 28- to 39-week–old nephritic mice 5 to 13 weeks anti-dsDNA positive (*n* = 13) were sacrificed as described previously.^3^ The kidneys and renal lymph nodes were isolated and processed for RNA isolation immunohistochemistry analysis as described.^5^

**Table 1:** Mice Used in This Study

Mouse no.	Age, wk	Time of Anti-dsDNA antibody Positivity, wk	Proteinuria uristix	ACR	Histology Score (range, 0–4)	TLS test result	RNA Sequencing
**1**	**7**	**0**	**0**	**12**	**2**	**Negative**	**M1**
**2**	**7**	**0**	**0**	**4**	**2**	**Negative**	**M2**
**3**	**7**	**0**	**0**	**32**	**2**	**Negative**	**M3**
**4**	**7**	**0**	**0**	**28**	**2**	**Negative**	**M4**
**5**	**7**	**0**	**0**	**42**	**2**	**Negative**	**M5**
6	35	5	4	2729	4	Positive	NP
7	35	7	0	2	2	Positive	NP
**8**	**29**	**4**	**0**	**2**	**2**	**Positive**	**M6**
**9**	**28**	**4**	**0**	**NP**	**2**	**Positive**	**M7**
**10**	**28**	**4**	**0**	**8**	**3**	**Positive**	**M8**
**11**	**29**	**4**	**0**	**61**	**3**	**Positive**	**M9**
12	20	7	0	61	2	Positive	NP
13	36	4	0	226	3	Positive	NP
14	36	4	0	108	3	Positive	NP
**15**	**26**	**5**	**0**	**7**	**2**	**Positive**	**M10**
16	37	13	4	NP	NP	Positive	NP
17	36	9	4	NP	4	Positive	NP
18	36	11	4	NP	NP	Positive	NP
19	30	5	4	NP	4	Positive	NP
20	41	14	2	NP	2	Positive	NP
21	30	Negative	4	NP	3	Positive	NP
22	38	8	4	NP	NP	Positive	NP
23	41	6	2	NP	NP	Positive	NP
24	41	13	2	NP	NP	Positive	NP
**25**	**39**	**13**	**4**	**NP**	**4**	**Positive**	**M11**
26	28	9	4	NP	NP	Positive	NP
**27**	**36**	**10**	**4**	**NP**	**4**	**Positive**	**M12**
**28**	**36**	**13**	**4**	**NP**	**3**	**Positive**	**M13**
**29**	**28**	**5**	**4**	**NP**	**4**	**Positive**	**M14**
**30**	**37**	**11**	**4**	**NP**	**4**	**Positive**	**M15**

Bold indicates selected for RNA sequencing.

ACR, albumin/creatinine ratio (normal, 0 to <30; microalbuminuria, 30 to ≤300; clinical proteinuria, >300); NP, not performed; TLS, tertiary lymphoid structures.

### Determination of Proteinuria, Anti-dsDNA Antibodies by Enzyme-Linked Immunosorbent Assay, and Histologic Classification of Lupus Nephritis

Urine samples were tested every week until the onset of proteinuria. Full-blown LN was defined when proteinuria reached 4+, as determined by Urine Stix (Bayer Diagnostics, Bridgend, UK): 0 to 1+ was defined as <1 g/L (physiologic proteinuria); 2+, ≥1 to 3 g/L; 3+, ≥3 to 20 g/L; and 4+, ≥20 g/L. End point urine were collected if possible and analyzed using the albumin/creatinine ratio (ACR) assay kit from PromoKine (Heidelberg, Germany). ACR results were defined as follows: normal, 0 to <30; microalbuminuria, 30 to ≤300; and clinical proteinuria, >300. However, chronic kidney disease may be present if ACR ≥30. Blood samples were taken every week until anti-dsDNA positivity was detected; thereafter, samples were taken every second week until proteinuria was detected. Serum samples were collected and stored at −20°C until use. Serum antibodies against dsDNA were determined by enzyme-linked immunosorbent assay as previously described.^23^^,^^24^ Sera were diluted twofold from 1/100 to 1/6400 in phosphate-buffered saline (PBS) (0.02% Tween), and the positive control 163c3 anti-dsDNA monoclonal antibody (kindly provided by T.N. Marion, The University of Tennessee Health Science Center, Memphis, TN^25^) was included in each enzyme-linked immunosorbent assay for assay validation and determination of cut-off value. The optical density cut-off values were set to >0.2 at A493, and positive titers were determined when 40% of positive control mAbs was reached. Classification of kidney damage was analyzed based on the 2019 European League Against Rheumatism and the American College of Rheumatology criteria.^26^ Pathologic findings were scored by observers who were blinded to the genotype. Paraffin-embedded sections 4 mm thick were dewaxed and stained with hematoxylin and eosin. The sections were scanned using a VS120 virtual slide microscope (Olympus, Asker, Norway). Each kidney was examined at ×400 magnification and scored from 0 to 4 based on the following features: glomerular size and hypercellularity, changes in glomerular matrix, and the degree of hypercellularity in the tubulointerstitium.

### Immunohistochemistry

Immunohistochemistry using Polink-2 Plus HRP detection kits for tissue (anti-rabbit and anti-rat) (Golden Bridge International Inc., Mukilteo, WA) was performed on Zink- or 4% paraformaldehyde-fixed kidneys embedded in paraffin. Anti-mouse CD3 was obtained from Dako (Glostrup, Denmark). Anti-mouse CD45R (B220) was purchased from R&D Systems (Minneapolis, MN). Antibodies against mouse B-cell lymphoma 6 (BCL6), CD21, and lymphotoxin β-receptor (LTBr) were purchased from Abcam (Cambridge, UK). Anti-mouse peripheral lymph node addressin (PNAD) and anti-mouse F4/80 were obtained from BioLegend (San Diego, CA). Anti-mouse monoclonal anti-DC antibody (MIDC)-8 was obtained from Nordic BioSite (Oslo, Norway). Images were collected with an Olympus microscope (BX51 and DP74).

### Immunofluorescence

Immunofluorescence staining was performed on 5-μm kidney cryosections. The sections were dried at room temperature for 30 minutes and then fixed for 5 minutes in 4% paraformaldehyde. Sections were washed three times in 1× PBS for 5 minutes each and incubated with blocking serum (1× PBS with 10% donkey serum) (AB7475, Abcam) for 30 minutes. The sections were incubated with primary antibodies [B220 and muscle, intestine and stomach expression (Mist) (BHLHA15, Biorbyt, Cambridge, UK) or B220 and forkhead box P3 (FoxP3) (Novus Biologicals, Bio-Techne Ltd., Abingdon, UK)] for 30 minutes and washed three times in 1× PBS for 5 minutes each. The sections were incubated with secondary antibody (AF488 anti-rat IgG and AF546 anti-rabbit IgG) for 30 minutes in the dark and were washed three times in 1× PBS for 5 minutes each. The sections were carefully dried and mounted using 10 μL of DAPI (Invitrogen, Fisher Scientific, Oslo, Norway). For each sample, a negative control was prepared by the same procedure with an irrelevant control antibody. Images were collected with an Olympus microscope (BX51 and DP74).

### Three-Dimensional Structure of TLS with Adjacent Arteries and Veins

Serial kidney sections (10-μm thick) from a NZB/W mouse were stained with hematoxylin, and images were obtained at ×4 magnification. The TLS, arteries, and veins were outlines, reconstructed, and visualized in three dimensions using the TrakEM2 plugin in ImageJ software version 1.52 (NIH, Bethesda, MD; *http://imagej.nih.gov/ij*).

#### Total RNA Extraction from Lymph Nodes, TLS, and Kidney Tissue

Total RNA of lymph nodes from 4- and 8-week–old and proteinuric mice, or immune aggregates/TLS dissected manually under the loupe (magnification, ×10) from proteinuric kidney, or kidney tissue from the same kidney, were purified by TRIzol regent (Ambion, Thermo Fisher, Oslo, Norway) according to manufacturer's instructions. Total RNA of lymph nodes from anti-dsDNA antibody–positive mice were isolated using miRNeasy mini kit (Qiagen, Oslo, Norway). The concentration and quality of extracted total RNA were assessed using the Agilent RNA 6000 nano kit with the Agilent 2100 Bioanalyzer instrument (Agilent, Matriks AS, Norway). All RNA used in this study had an RNA integrity number ≥7.

#### Total RNA Extraction from Total Kidney

Total RNA samples were prepared for sequencing using the Qiagen Allprep total RNA kit (Qiagen). Briefly, tissue sections (20 to 30 μg) taken from the middle of the kidneys were lyzed and homogenized in 600 μL of Buffer RLT in 2 mL of Magna Lyser green beads tubes (Roche Life Sciences, Oslo, Norway) at 600 × *g* for 30 seconds using the Precellys 24 tissue homogenizer (Bertin Technologies, Aix-en-Provence, France). The lysates were centrifuged at full speed for 3 minutes, the supernatant was carefully transferred to the AllPrep spin column, and RNA isolation was performed according to the manufacturer's protocol. The isolated RNA was analyzed by Agilent. Only RNA samples of high quality (RNA integrity number ≥8.0) were used. Sequencing of total kidney mRNA was performed by Eurofins Genomics (Ebersberg, Germany) using the Illumina HiSeq 2000 system.

### Sequencing of Paired Lymph Nodes, TLS, and Kidneys

#### Library Preparation

Polyadenylated mRNA was isolated from total RNA using Dynabeads mRNA Direct Micro Kit (Ambion, Thermo Fisher) followed by its fragmentation using RNase III with mean sizes of 100 to 200 nt. The fragmented mRNA was cleaned and the yield and size distribution determined using the Agilent RNA 6000 Pico kit with the Agilent 2100 Bioanalyzer instrument. After adapter ligation at both ends of fragmented mRNA, reverse transcription was performed, followed by PCR amplification. After purification of amplified cDNA, the yield and size distribution were analyzed using an Agilent High Sensitivity DNS kit with Agilent 2100 Bioanalyzer instrument.

#### Sequencing

All libraries were amplified with emulsion PCR (Ion One Touch 2 instrument), and enrichment of template Ion Sphere Particles was performed with the Ion One Touch ES system. Quality control of Ion Sphere Particles was performed with an Ionsphere Quality Control kit using Qubit version 2.0 (Life Technologies, Thermo Fisher, Oslo, Norway). The enriched Ion Sphere Particles were sequenced using an Ion Torrent 316 chip with sequencer Ion Torrent Personal Genome Machine (Ion Torrent PGM) according to manufacturer's instructions.

#### Bioinformatics Analysis

All sequences were imported and analyzed in CLC Genomics Workbench 8 (CLCbio, Aarhus Denmark). Adaptor sequences were trimmed, and RNA sequencing was conducted using Mus_musculus GRCm 38.80 as a reference genome by default.

### Deep Sequencing of Whole Kidney Sections

#### RNA Sequencing

RNA sequencing cDNA libraries were prepared from the total RNA isolated from kidneys of 15 NZB/W F1 mice (Table 1). Fifteen 3′-fragments with an insert size of approximately 200 to 450 bp were prepared and sequenced using the Illumina HiSeq 2000 version 3.0 system (Eurofins Genomics). The samples were divided into three channels with five libraries per channel containing 1 × 100-bp single-read module. The sequences were demultiplexed according to the 6-bp index code allowing 1 mismatch (Eurofins Genomics).

#### Alignments and Assignment of Reads to Genes

Eurofins Genomics performed alignments and assignment of reads to genes. The alignment of reads to a reference sequence was performed using the BWA-backtrack version 0.6.2-r126 (*http://bio-bwa.sourceforge.net*, last accessed June 18, 2019). Raw read counts were created using HTSeq with Python software version 2.7 (Python Software Foundation, Fredericksburg, VA; *https://www.python.org*). Reads with unique mapping positions were considered for read counting. Paired-end reads that were mapped to the same reference with approximately the expected insert size were counted as one read. Paired-end reads that were mapped to different references or with an unexpected insert size were counted as two reads. If only one read of a pair was mapped, it was counted as one read. Only reads that overlapped exon features were counted. All reads mapping to features with the same identifier were summed. The gene attribute was used as feature identifier. Reads mapping to multiple features with different identifier were ignored for read counting. The mean read length was 100.0 (Eurofins Genomics).

#### Filtering and Normalization for Composition Bias

The CPM (counts per million) function from the *edgeR* library was used to generate the CPM values; the CPM values were further filtered. The ratio of RNA production was estimated by using a weighted trimmed mean of log expression ratios called the trimmed mean of M values. The *calcNormFactors* function from *edgeR* package calculated the normalization factors among libraries. These normalization factors are rescaled by the mean of the normalized library sizes. Normalized read counts were obtained by dividing raw read counts by these rescaled normalization factors. This was performed to eliminate composition biases among libraries.^27^ A multidimensional scaling plot was generated with the plotMDS function from limma package. Distances between samples was calculated with leading fold change defined as the root mean square of the largest 500 log2 fold changes among samples.

#### Differential Expression Analysis

Differential expression analysis was performed using the edgeR package.^28^ Differential expression data were filtered to contain a false discovery rate <0.05. An empirical Bayes procedure was used to shrink the dispersions toward a consensus value, borrowing information among genes. The results were tested for differential expression using the generalized linear model likelihood ratio test. The likelihood ratio test was performed by estimating two groups and by comparing the fit of one group with the fit of the other. To deal with multiple tests, individual tests were made with separate computations to test for different contrasts. Three different contrasts were made to test the hypothesis that the different coefficients in each contrast (ie, the three different groups) were equal. The glmLRT function from edgeR was used to conduct likelihood ratio tests for the coefficients in the linear model, such as the Fisher exact test, and adapted for over dispersed data.^27^ The results were corrected for multiple hypothesis testing via the Benjamini-Hochberg procedure. For each gene, an adjusted *P* value was calculated to enable the expected proportion of positive results returned that were false-positive results (ie, false discovery rate). Venn diagrams were drawn to visualize the overlap between up-regulated and down-regulated genes among all three groups using the vennDiagram package from limma. All relevant data have been deposited in the Gene Expression Omnibus (*https://www.ncbi.nlm.nih.gov*; accession number GSE155405).

### Statistical Analysis

GraphPad Prism software version 5.0 (GraphPad Software, San Diego, CA) was used to perform statistical comparison analyses. Results are expressed as means ± SEM. Statistical significance was set at *P* < 0.05. Statistical significance was assessed using a paired *t*-test, one-way or two-way analysis of variance, followed by the Bonferroni posttest as indicated in the figures. Differentially expressed (DE) genes were divided into up-regulated and down-regulated genes, according to an adjusted *P* <0.05 and logFC value 1.0/−1.0, respectively. Venn diagrams were drawn to visualize the overlapping of DE genes using the groupings from the multidimensional scaling plot plot. Statistical significance is indicated as follows in the figures. A Spearman's correlation matrix was generated based on clinical parameters and gene expression.

## Results

### TLS Occur Close to the Pelvic Wall, Large Arteries, and Large Veins and Are Organized into Large Interconnected Networks of Immune Aggregates within the Kidneys of Lupus-Prone Mice

**Figure 1:**
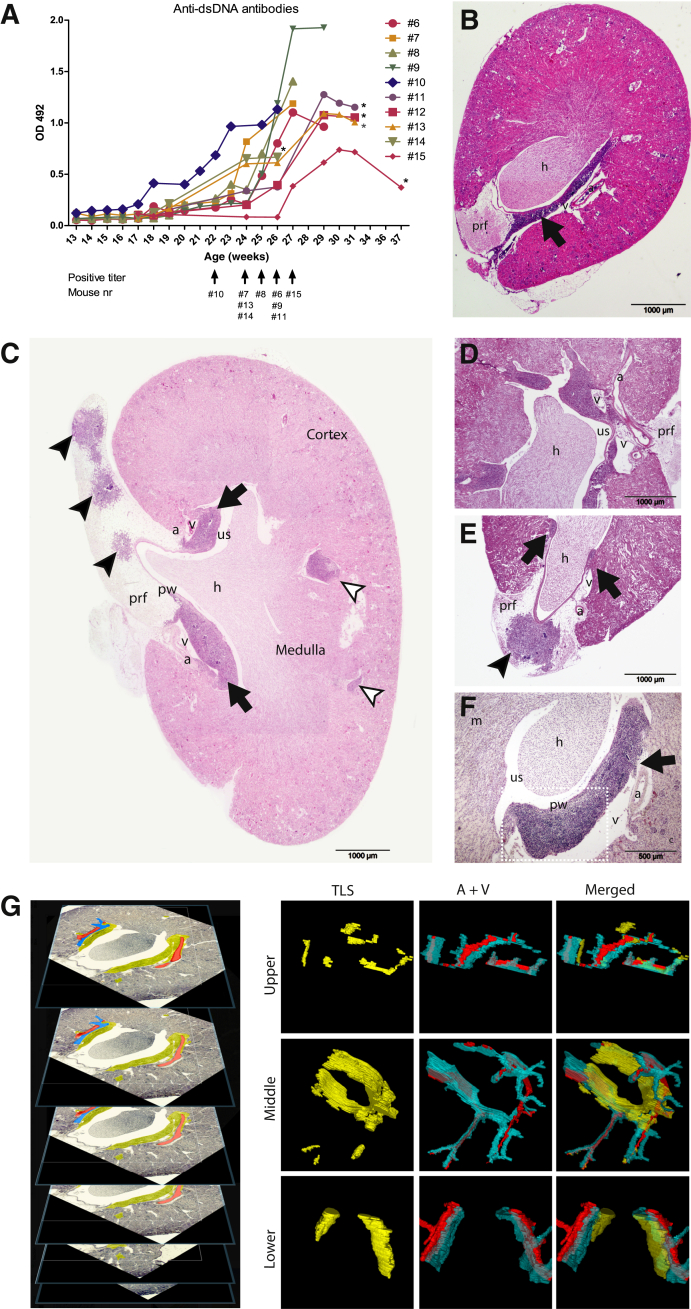
All mice with anti–double-stranded DNA (dsDNA) antibody production for 4 to 5 weeks had large tertiary lymphoid structures (TLS) within the pelvic area connected to the biggest veins and arteries. **A:** The anti-dsDNA antibody production in sera was followed by an in-house enzyme-linked immunosorbent assay. The absorbance was measured at OD 492, and antibody titer was calculated based on 40% of the positive control. The figure shows the results of the mice later selected for deep sequencing (M6-M15) (Table 1). **Asterisks** indicate proteinuric mice (group 3). **B** and **F:** Transverse sections of kidneys revealed large TLS (**black arrows**) in connection to the pelvic wall and close to the biggest veins and arteries. **C** and **D:** Longitudinal sections present several areas with immune aggregates organized as TLS. **C** and **E:** TLS (**black arrows**) are also detected in the perirenal fat (**black arrowheads**) and in the cortex of proteinuric mice (**white arrowheads**). **G:** A kidney from a proteinuric mouse divided into three parts was serial sectioned and stained with eosin. The TLS, largest veins, and largest arteries were depicted in ImageJ software version 1.52 and assembled as a three-dimensional structure. The sections from the kidney were highlighted for TLS (yellow), arteries (red), and veins (blue), and the layers were merged into three figures depicting the upper, middle, and lower parts of the kidney. Stippled squares in **panel F** depict area of interest in Figure 2A. The images are representative of group 2 and group 3 mice. Scale bars: 1000 (**B**–**E**); 500 μm (**F**). Original magnification, ×4 (**G**). a, artery; h, hilum; m, medulla; prf, perirenal fat; pw, pelvic wall; us, urinary space; v, vein.

The production of anti-dsDNA antibody was measured every week until antibody-positive titer and then every second week until onset of proteinuria or until the mice reached 4 to 5 weeks with antibody positivity (Figure 1A and Table 1). All mice with anti-dsDNA antibody-positive titer for 4 to 5 weeks had developed TLS near the pelvic wall, large arteries, and large veins (Figure 1, B–E). Some mice had TLS within the perirenal fat (Figure 1, C and E). Smaller TLS were also detected in the cortex of kidneys from proteinuric mice (Figure 1, C and F). To examine the structural specifications of TLS and to investigate whether the TLS developed in separate regions of the kidneys, a three-dimensional structure of serial sections of a whole kidney was made (Figure 1G). The three-dimensional structure revealed an interconnected large network of immune aggregates located close to or surrounding the arteries and veins (Figure 1G). The larger structures were found in the renal pelvic area between the pelvic wall and the biggest veins (Figure 1G), whereas the smaller structures were located around the arteries in the superior and inferior sections of the kidney (Figure 1G).

### TLS Contain All Cells Needed To Be a Functional Immunologic Site for Activation and Regulation of Immune Cells

**Figure 2:**
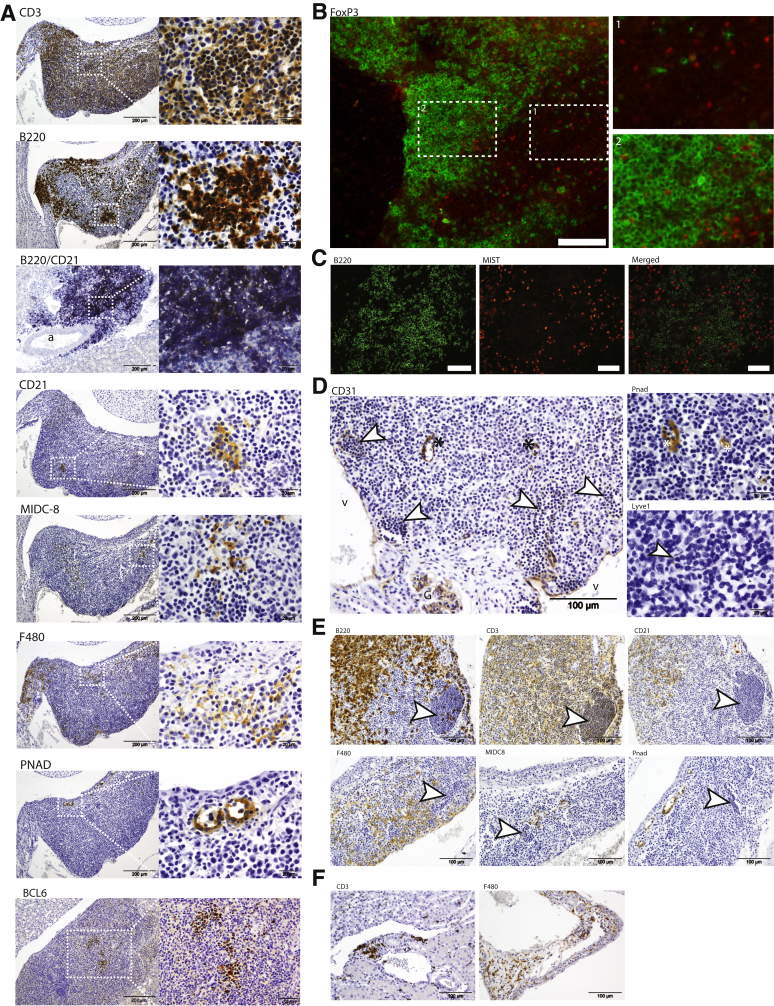
T cells and B cells are prominent cells of tertiary lymphoid structures (TLS) and are organized into distinct T- and B-cell areas with a large network of blood and lymph vessels. **A:** Immunohistochemistry performed with anti-CD3 detecting T cells, anti-B220 detecting B cells, anti-CD21 detecting follicular dendritic cells (FDCs), double staining B220 (purple) and CD21 (brown), anti-F4/80 detecting macrophages, anti–peripheral lymph node addressin (PNAD) (MECA-79) detecting high endothelial venules (HEVs), anti–monoclonal anti–dendritic cell (DC) antibody (MIDC)-8 detecting activated DCs, and anti–B-cell lymphoma 6 (BCL6) detecting germinal center B and T cells. **B:** Immunofluorescence performed with anti–forkhead box P3 (FoxP3) detecting regulatory T cells and anti-B220 detecting B cells. **C:** Immunofluorescence performed with anti-B220 detecting B cells and anti– muscle, intestine and stomach expression (Mist)-1 detecting plasma cells. **D:** CD31 staining detecting endothelial cells reveals a network of different vessels with positive wispy cells in larger vessels (**white arrowheads**) and microcapillaries or thicker cells as in HEVs (**black asterisk**). The HEVs stained positively for PNAD (**white asterisks**), and some of the thin vessels are lymphatic vessel endothelial hyaluronan receptor 1 positive. **E:** The larger thin vessels (**white arrowheads**) are filled with leukocytes that were mostly CD3-positive T cells and B220-positive B cells and a few MIDC-8–positive DCs. None of the immune cells within the vessels were F4/80 or CD21-positive macrophages and FDCs, respectively. **F:** In young anti–double-stranded DNA–negative mice, CD3-positive T cells and F480-positive macrophages could be detected within the pelvic wall. Scale bars: 200 μm (**A**, **left column**); 100 μm (**B**; **D**, **left column**, **E**, and **F**); 20 μm (**A** and **D**, **right columns**); 50 μm (**C**). Original magnification: ×10 (**A**, **left column**); ×20 (**B**, and **D**, **left column**); ×60 (**A**, **right column**, and **C**). G, glomerulus; v, vein.

Most cells in TLS were CD3^+^ T cells with surrounding characteristic B220^+^ B-cell areas (Figure 2A). The B-cell areas contained a network of CD21^+^ follicular DCs (FDCs) (Figure 2A), and the T-cell areas were rich in activated MIDC-8^+^ DCs (Figure 2A8) and a few FoxP3^+^ cells (Figure 2B). F4/80^+^ macrophages were detected within the pelvic wall, in the TLS close to the kidney tissue or perirenal fat, and interspersed within the structure (Figure 2A). PNAD and high endothelial venules (HEVs) were detected within the pelvic wall (Figure 2A). The presence of germinal centers (BCL6) was detected in larger TLS (Figure 2A). Mist and plasma cells were detected within the TLS in areas outside the dense B-cell zones and germinal centers (Figure 2C). In the large aggregates, many small microvessels were detected throughout the structure, whereas HEVs were mostly located along the pelvic wall (Figure 2D). Large lymphatic vessel endothelial hyaluronan receptor 1–positive thin vessels were identified and contained CD3^+^ and B220^+^ T and B cells, in addition to a few MIDC-8^+^ DCs (Figure 2E). Previously obtained animals (*n* = 49^3^^,^^5^) were analyzed for the presence of TLS, and a high correlation between the anti-dsDNA antibody titer and development of TLS was observed (Table 2). Even in mice negative for anti-dsDNA antibody, small aggregates of CD3^+^ T cells and F4/80^+^ macrophages could be observed within the pelvic wall of the kidneys (Figure 2F).

**Table 2:** Spearman Correlation Matrix of Real-Time Quantitative PCR Result and Clinical Parameters

	Age	Weeks of Anti-dsDNA Antibody	Titer	Proteinura	ACR	Histologic Score	*Ltb*	*Ltbr*	RANKL	*Ccl21a*	*Cxcl13*	*CCR7*	Aid	*Vcam1*	*Icam1*	*Pou2af1*	Glycam
Age		**0.902**	**0.895**	**0.625**	0.142	**0.789**	**0.726**	0.430	**0.574**	0.324	**0.794**	**0.801**	**0.658**	**0.828**	**0.885**	**0.761**	**0.682**
Weeks of anti-dsDNA antibody	**<0.001**		**0.796**	**0.646**	0.302	**0.788**	**0.775**	**0.631**	**0.549**	**0.529**	**0.762**	**0.877**	**0.681**	**0.844**	**0.905**	**0.729**	**0.784**
Titer	**<0.001**	**<0.001**		0.360	0.209	**0.678**	**0.805**	0.186	**0.752**	0.324	**0.855**	**0.831**	**0.814**	**0.733**	**0.778**	**0.783**	**0.630**
Proteinura	**0.013**	**0.009**	0.187		0.502	**0.845**	0.244	**0.768**	-0.070	0.244	0.279	**0.558**	0.035	**0.768**	**0.713**	0.367	0.489
ACR	0.677	0.366	0.537	0.115		0.466	0.342	0.306	0.064	**0.790**	0.215	0.466	0.073	0.479	0.098	0.368	0.393
Histologic score	**<0.001**	**<0.001**	**0.006**	<0.001	0.148		**0.637**	**0.562**	0.318	0.442	**0.651**	**0.826**	0.425	**0.908**	**0.893**	**0.653**	**0.647**
*Ltb*	**0.002**	**0.001**	**<0.001**	0.380	0.303	**0.011**		0.171	**0.875**	**0.575**	**0.914**	**0.814**	**0.893**	**0.736**	**0.780**	**0.903**	**0.829**
*Ltbr*	0.110	**0.012**	0.507	**0.001**	0.360	**0.029**	0.541		-0.089	0.254	0.175	0.457	0.107	**0.543**	0.486	0.216	0.393
Rankl	**0.025**	**0.034**	**0.001**	0.805	0.852	0.247	**<0.001**	0.752		0.229	**0.818**	**0.621**	**0.929**	0.454	**0.560**	**0.806**	**0.664**
*Ccl21a*	0.239	**0.043**	0.238	0.380	**0.004**	0.099	**0.025**	0.362	0.413		0.450	**0.518**	0.311	**0.543**	0.451	0.463	0.504
*Cxcl13*	**<0.001**	**0.001**	**<0.001**	0.314	0.526	**0.009**	**<0.001**	0.533	**<0.001**	**0.092**		**0.825**	**0.854**	**0.782**	**0.846**	**0.888**	**0.704**
*Ccr7*	**<0.001**	**<0.001**	**<0.001**	**0.031**	0.149	**<0.001**	**<0.001**	0.087	**0.013**	**0.048**	**<0.001**		**0.675**	**0.846**	**0.886**	**0.767**	**0.704**
Aid	**0.008**	**0.005**	**<0.001**	0.902	0.831	0.115	**<0.001**	0.704	**<0.001**	0.260	**<0.001**	**0.006**		**0.536**	**0.635**	**0.806**	**0.711**
*Vcam1*	**<0.001**	**<0.001**	**0.002**	**0.001**	0.136	**<0.001**	**0.002**	**0.037**	0.089	**0.037**	**0.001**	**<0.001**	**0.040**		**0.925**	**0.797**	**0.768**
*Icam1*	**<0.001**	**<0.001**	**0.001**	**0.004**	0.789	**<0.001**	**0.001**	0.078	**0.037**	0.106	**<0.001**	**<0.001**	**0.015**	**<0.001**		**0.815**	**0.780**
*Pou2af1*	**0.001**	**0.002**	**0.001**	0.179	0.265	**0.008**	**<0.001**	0.439	**<0.001**	0.082	**<0.001**	**0.001**	**<0.001**	**<0.001**	**<0.001**		**0.883**
Glycam	**0.005**	**0.001**	**0.012**	0.065	0.232	**0.009**	**<0.001**	0.147	**0.007**	0.056	**0.003**	**0.003**	**0.003**	**0.001**	**0.001**	**<0.001**	

Bold indicates significantly correlated at *P* < 0.05. ACR, albumin/creatinine ratio; dsDNA, double-stranded DNA.

### Comparison of DE Genes in Isolated TLS, Kidney Tissues, and Lymph Nodes Reveals Similarities between Lymph Nodes and TLS

**Figure 3:**
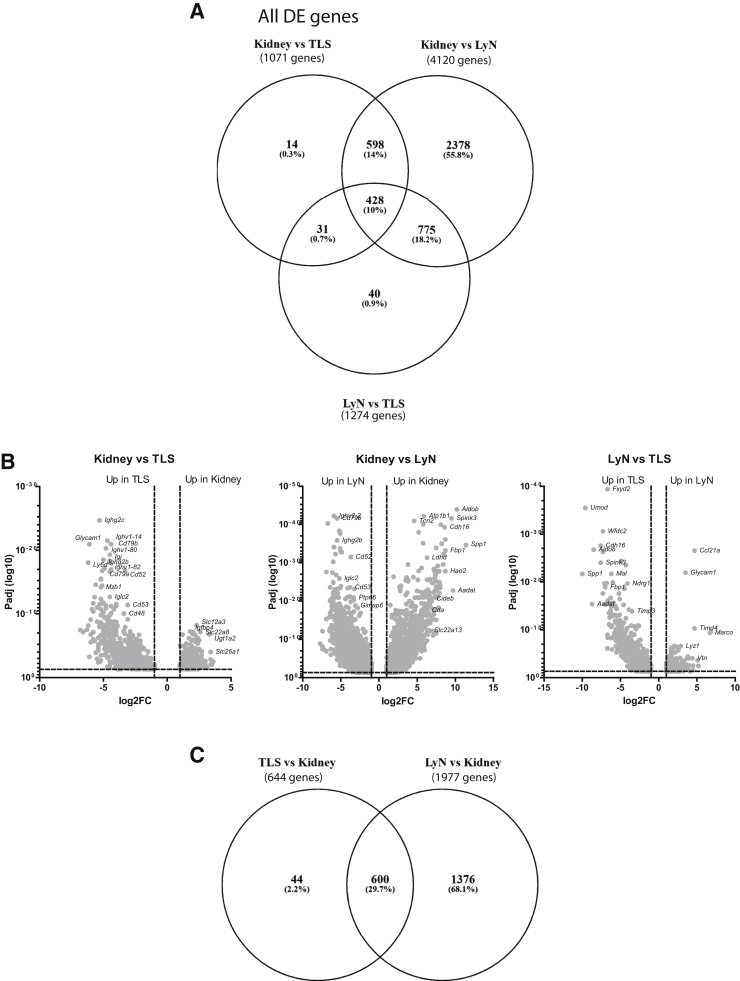
Gene expression in tertiary lymphoid structures (TLS) resembles lymph nodes (LyN). **A:** Venn diagram depicting the common and unique genes of all differentially expressed (DE) genes in kidney versus lymph nodes, kidney versus TLS, and lymph nodes versus TLS. **B:** Volcano plot showing up- and down-regulated gene expression in kidney versus lymph nodes, kidney versus TLS, and lymph nodes versus TLS comparisons. **C:** Venn diagram of 644 TLS genes compared with 1977 lymph nodes genes showing 600 common genes. FC, fold change; LyN, lymph nodes, Padj, *P* adjusted.

To investigate whether the TLS resembles lymph nodes with respect to gene expression, total RNA was isolated from renal draining lymph nodes, kidney, and TLS dissected from kidney (*n* = 3). The gene expression was analyzed by the Ion Torrent PGM system, and 13,982 DE genes were identified in a comparison of genes between TLS and kidney, lymph nodes versus kidney, and TLS versus lymph nodes (Supplemental Table S1). Figure 3A shows a Venn diagram of the significantly DE genes in the different comparisons (Supplemental Tables S2–S4). Kidney versus TLS reveled 1071 DE genes, where 371 genes were up-regulated in kidney and 644 genes were up-regulated in TLS (Figure 3, A and B, and Supplemental Table S2), whereas kidney versus lymph nodes had the most DE genes (Figure 3, A and B, and Supplemental Table S3). Comparing the 644 genes up-regulated in TLS with the genes significantly up-regulated in lymph nodes compared with kidney (Supplemental Table S3) revealed 600 common genes (Figure 3C). Reactome pathway analyzer recognized 400 of these genes, and Table 3 lists the top 20 enriched pathways. Search Tool for the Retrieval of Interacting Genes/Proteins (STRING) functional enrichment analyses recognized 455 genes found in gene ontology pathways mostly involved in activation and regulation of the immune system (Table 4). Of interest, 62 TLS genes were found in the Gene Ontology term *hematopoietic or lymphoid organ development* (Table 4). DE genes between TLS and lymph nodes defined the kidney-specific genes found in isolated TLS (Figure 3B and Supplemental Table S4).

**Table 3:** Tertiary Lymphoid Structure Genes Found Enriched in Pathways Recognized by the Reactome Pathway Analyzer

Pathway	Entities					Reactions		
	Found, *n*	Total, *n*	Ratio	*P*	FDR	Found, *n*	Total, *n*	Ratio
CD22-mediated BCR regulation	68	104	0.009	1.11 × 10^16^	1.59 × 10^14^	4	4	0.001
Classic antibody-mediated complement activation	68	108	0.01	1.11 × 10^16^	1.59 × 10^14^	2	2	0
FCGR activation	70	116	0.01	1.11 × 10^16^	1.59 × 10^14^	5	5	0.001
Antigen activates BCR, leading to generation of second messengers	75	127	0.011	1.11 × 10^16^	1.59 × 10^14^	10	14	0.002
Creation of C4 and C2 activators	68	119	0.011	1.11 × 10^16^	1.59 × 10^14^	2	6	0.001
Initial triggering of complement	70	128	0.012	4.44 × 10^16^	5.28 × 10^14^	8	19	0.002
FCERI mediated Ca^+^2 mobilization	72	135	0.012	5.55 × 10^16^	5.66 × 10^14^	5	6	0.001
Role of LAT2, NTAL, and LAB on calcium mobilization	66	117	0.011	7.77 × 10^16^	6.92 × 10^14^	4	5	0.001
FCERI-mediated MAPK activation	70	134	0.012	4.11 × 10^15^	3.25 × 10^13^	12	19	0.002
Role of phospholipids in phagocytosis	70	135	0.012	5.77 × 10^15^	4.1 × 10^13^	6	9	0.001
Regulation of complement cascade	70	141	0.013	4.46 × 10^14^	2.9 × 10^12^	5	23	0.003
Scavenging of heme from plasma	64	125	0.011	1.45 × 10^13^	8.57 × 10^12^	1	12	0.002
Regulation of actin dynamics for phagocytic cup formation	69	143	0.013	2.47 × 10^13^	1.36 × 10^11^	6	11	0.001
Immunoregulatory interactions between a lymphoid and a nonlymphoid cell	90	222	0.02	9.56 × 10^13^	4.88 × 10^11^	13	26	0.003
Complement cascade	71	156	0.014	1.6 × 10^12^	7.52 × 10^11^	15	50	0.006
Signaling by the BCR	84	209	0.019	8.4 × 10^12^	3.7 × 10^10^	23	28	0.004
FCGR-dependent phagocytosis	73	172	0.016	1.82 × 10^11^	7.66 × 10^10^	17	25	0.003
Binding and uptake of ligands by scavenger receptors	65	168	0.015	7.84 × 10^9^	3.06 × 10^7^	2	22	0.003
FCERI-mediated NF-κB activation	68	181	0.016	1.09 × 10^8^	4.05 × 10^7^	14	17	0.002
Cell surface interactions at the vascular wall	79	228	0.021	2.22 × 10^8^	7.78 × 10^7^	18	58	0.007
FCERI signaling	78	235	0.021	1.49 × 10^7^	5.05 × 10^6^	40	52	0.007
PD-1 signaling	16	26	0.002	2.28 × 10^5^	7.31 × 10^4^	4	4	0.001
Generation of second messenger molecules	17	32	0.003	7.74 × 10^5^	2.31 × 10^3^	13	14	0.002
Translocation of ZAP-70 to immunological synapse	14	23	0.002	8.09 × 10^5^	2.31 × 10^3^	4	4	0.001
Phosphorylation of CD3 and TCR ζ chains	15	26	0.002	8.24 × 10^5^	2.31 × 10^3^	7	7	0.001

Data from *https://reactome.org*, last accessed June 25, 2020.

BCR, B-cell receptor; FCERI, Fcε receptor; FCGR, Fcγ receptor; FDR, false discovery rate; LAB, linker for activation of B cells; LAT2, linker for activation of T cells 2; MAPK, mitogen-activated protein kinase; NTAL, non–T-cell activation linker; PD-1, programmed death 1; TCR, T-cell receptor; ZAP-70, ζ chain–associated protein kinase 70.

**Table 4:** Tertiary Lymphoid Structure Genes Were Enriched Following GO Pathways

GO no.	Term Description	Observed Gene Count	Background Gene Count	False Discovery Rate
0002376	Immune system process	198	1703	8.07 × 10^89^
0006955	Immune response	139	914	1.10 × 10^71^
0002682	Regulation of immune system process	139	1165	9.67 × 10^60^
0002684	Positive regulation of immune system process	104	771	1.79 × 10^47^
0001775	Cell activation	86	552	6.31 × 10^43^
0006952	Defense response	114	1079	6.31 × 10^43^
0045321	Leukocyte activation	80	464	1.15 × 10^42^
0050776	Regulation of immune response	89	635	2.09 × 10^41^
0050896	Response to stimulus	280	6616	3.33 × 10^40^
0046649	Lymphocyte activation	69	378	6.73 × 10^38^
0050778	Positive regulation of immune response	71	438	4.06 × 10^36^
0051249	Regulation of lymphocyte activation	68	396	6.63 × 10^36^
0002694	Regulation of leukocyte activation	71	479	6.15 × 10^34^
0050865	Regulation of cell activation	72	520	9.35 × 10^33^
0002250	Adaptive immune response	54	260	8.79 × 10^32^
0045087	Innate immune response	71	534	2.61 × 10^31^
0002252	Immune effector process	59	395	4.62 × 10^28^
0050863	Regulation of T cell activation	52	287	4.62 × 10^28^
1903037	Regulation of leukocyte cell-cell adhesion	50	264	9.52 × 10^28^
0032944	Regulation of mononuclear cell proliferation	46	217	2.90 × 10^27^
0006950	Response to stress	155	2899	4.47 × 10^27^
0048583	Regulation of response to stimulus	174	3552	6.66 × 10^27^
0050670	Regulation of lymphocyte proliferation	45	215	1.64 × 10^26^
0048584	Positive regulation of response to stimulus	122	1922	1.76 × 10^26^
0051707	Response to other organism	76	779	8.22 × 10^26^
0022407	Regulation of cell-cell adhesion	54	360	8.45 × 10^26^
1903039	Positive regulation of leukocyte cell-cell adhesion	42	190	1.50 × 10^25^
0050870	Positive regulation of T-cell activation	41	180	2.31 × 10^25^
0051251	Positive regulation of lymphocyte activation	46	253	6.07 × 10^25^
0009605	Response to external stimulus	113	1794	4.76 × 10^24^
0022409	Positive regulation of cell-cell adhesion	43	227	6.74 × 10^24^
0042110	T-cell activation	44	244	1.00 × 10^23^
0002253	Activation of immune response	42	221	2.18 × 10^23^
0030155	Regulation of cell adhesion	65	624	2.39 × 10^23^
0050867	Positive regulation of cell activation	48	314	3.13 × 10^23^
0002696	Positive regulation of leukocyte activation	47	300	3.88 × 10^23^
0050789	Regulation of biological process	308	9594	7.55 × 10^23^
0007165	Signal transduction	164	3594	7.24 × 10^22^
0051716	Cellular response to stimulus	205	5142	7.68 × 10^22^
0065007	Biological regulation	317	10,168	8.13 × 10^22^
0098542	Defense response to other organism	50	397	6.23 × 10^21^
0002520	Immune system development	64	687	1.20 × 10^20^
0002757	Immune response–activating signal transduction	35	168	1.46 × 10^20^
0002764	Immune response–regulating signaling pathway	36	184	2.22 × 10^20^
0002521	Leukocyte differentiation	46	340	2.41 × 10^20^
0030098	Lymphocyte differentiation	40	243	2.55 × 10^20^
0048518	Positive regulation of biological process	206	5340	2.81 × 10^20^
0048534	Hematopoietic or lymphoid organ development	62	659	3.29 × 10^20^
0045785	Positive regulation of cell adhesion	48	383	4.89 × 10^20^
0002376	Immune system process	198	1703	8.07 × 10^89^

Data from *http://geneontology.org/docs/go-enrichment-analysis*, last accessed June 25, 2020.

GO, Gene Ontology.

### Gene Expression in Total Kidney Reflects Development of TLS and Progression of LN

**Figure 4:**
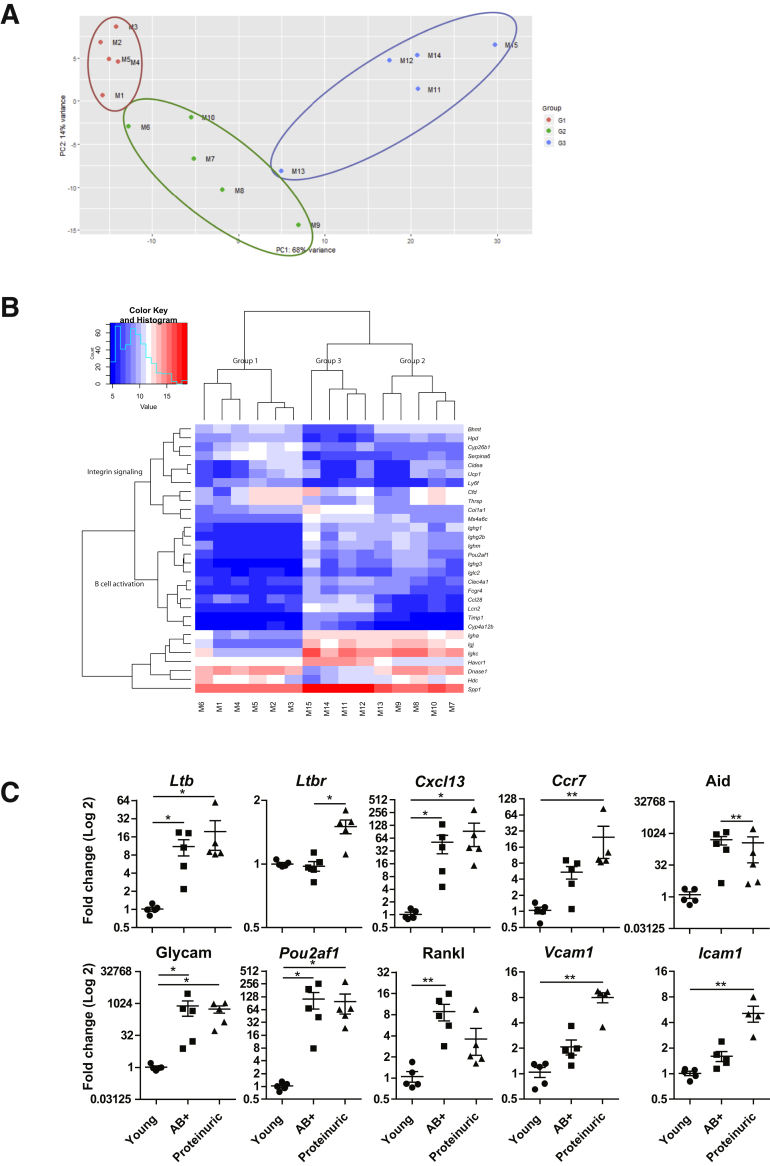
The gene expression analyses of total kidneys from the selected New Zealand black × New Zealand white (NZB/W) mice clustered into three groups. **A:** Multidimensional scaling plot showing the relationship among all mice kidney samples (M1 to M15). Group 1 included 7-week–old (M1 to M5), group 2 included antibody positive (AB^+^) mice (M6 to M10), and group 3 included proteinuric mice (M11 to M15). **B:** Heatmap of the 30 most variable genes across all samples. The dendrogram shows a global relationship between samples and genes. The *z* score indicates whether the genes are above or below the mean and by how many SDs. **C:** Real-time quantitative PCR of *Ltb*, *Ltbr*, *Cxcl13*, *Ccr7*, Aid (alias *Aicda*), *Pou2af1*, Rankl (alias *Tnfsf11*), Glycam (*Glycam1*), *Vcam1*, and *Icam1* gene expression analyzed on the cDNA from group 1 (young), group 2 (AB^+^), and group 3 (proteinuric) mice. ∗*P* < 0.05, ∗∗*P* < 0.01.

To investigate the gene expression profile during the development of TLS within the kidneys of lupus-prone mice during LN progression, RNA sequencing was performed on whole kidneys isolated from three groups of mice; young, antibody negative (group 1), 4 to 5 week anti-dsDNA antibody-positive mice (group 2), and proteinuric mice (group 3). Five mice in each group were selected based on their age (7 weeks old), weeks of anti-dsDNA antibody-positive serum samples (4 to 5 weeks), and proteinuria (+3 and +4) and renamed (Table 1 and Figure 1A). A two-dimensional scatterplot determining the greatest sources of variation between samples with approximations of log2-fold changes revealed that one group 3 mouse (M13) clustered close to group 2 mice and one group 2 mouse (M6) clustered close to group 1 mice (Figure 4A). This finding was confirmed in a heatmap presenting the top 30 variable (up- and down-regulated) genes of the sequencing data sets where the mice clustered into three distinct branches (Figure 4B). Most of these genes are involved in the immune system and specifically to integrin signaling and B-cell activation. Of interest, among the highly variable genes are genes associated with the formation of TLS [*Ighg1*, *Ighg2*, Igj (*Jchain*), *Ighm*, Igha, *Ighg3*, *Iglc2*, *Igkc*, *Pou2af1*].

A selection of genes important for TLS induction were analyzed by real-time quantitative PCR. The genes *Ltb*, *Cxcl13*, Aid (alias *Aicda*), *Pou2af1*, Rankl (alias *Tnfsf11*), and Glycam (alias *Glycam1*) were significantly up-regulated in group 2 mice compared with group 1 mice (Figure 4C). The genes *Ltbr*, *Ccr7*, *Pou2af1*, Glycam, *Vcam1*, and *Icam1* were significantly up-regulated in group 3 mice (Figure 4C). The expression of these genes were positively correlated to anti-dsDNA antibody production and TLS formation (Table 2).

**Figure 5:**
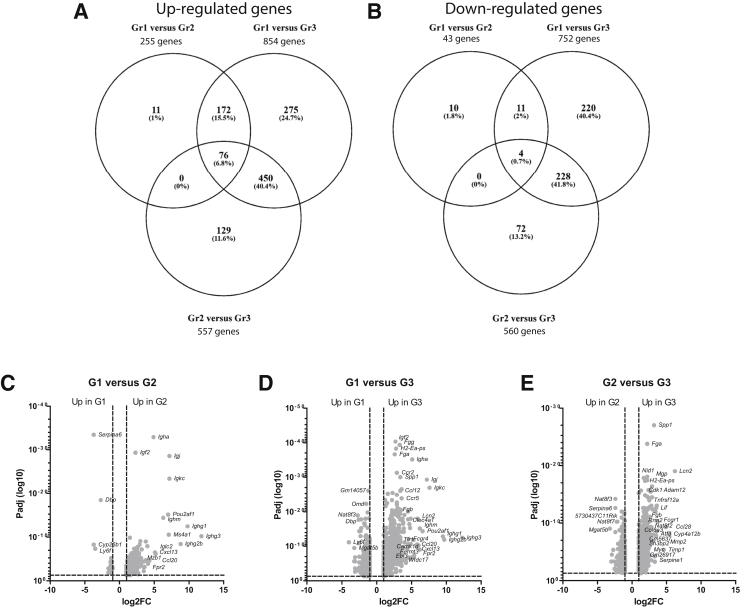
Gene expression of total kidneys reflect development of tertiary lymphoid structures. Venn diagram of up-regulated (**A**) and down-regulated (**B**) genes in group 1, 2, and 3 mice. Volcano plot depicting the gene expression in group 1 versus group 2 (**C**), group 1 versus group 3 (**D**), and group 2 versus group 3 (**E**) comparisons. Group 1 (Gr1, alias G1), young, antibody negative mice; group 2 (Gr2, alias G2), 4 to 5 week anti-dsDNA antibody-positive mice; group 3 (Gr3, alias G3), proteinuric mice.

**Figure 6:**
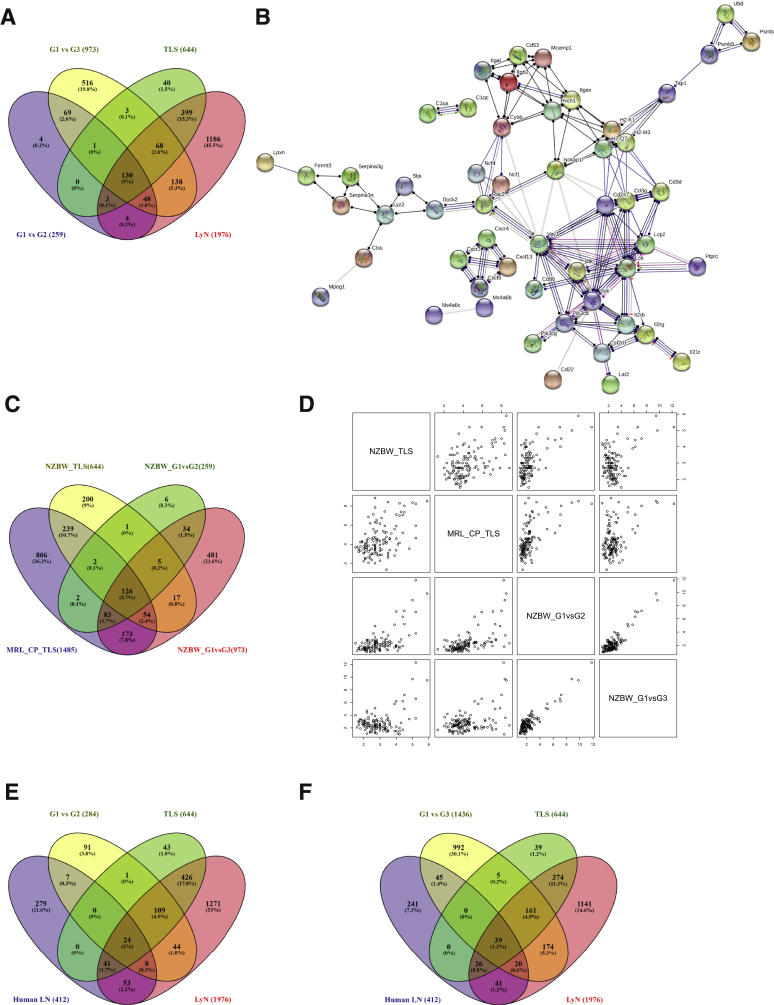
Tertiary lymphoid structure (TLS)-related genes are up-regulated in murine and human lupus nephritis kidneys. **A:** Venn diagram of group 2 and 3 gene expression compared with gene expression in TLS and lymph nodes (LyN). **B:** Search Tool for the Retrieval of Interacting Genes/Proteins interaction network of the 133 common genes showing the molecular action with the minimum required interaction score of highest confidence (0.900). The disconnected nodes are not shown. Action types are as follows: green, activation; red, inhibition; blue, binding; purple, catalyst; pink, posttranslational modification; and black, reaction. Action effects are as follows: arrow indicates positive; red line, negative; dot, unspecified. **C:** Venn diagram showing the number of differentially expressed (DE) genes in New Zealand black × New Zealand white (NZB/W) kidney TLS (*n* = 644), DE up-regulated genes in group 2 versus group 1 (*n* = 259), DE up-regulated genes in group 3 versus group 1 (*n* = 973), and the 1485 up-regulated genes found in the chorus plexus TLS of MRL lpr/lpr mice. **D:** Paired correlation plots on scaled log2 fold change values of the 126 common genes found in **panel C**. **E:** Venn diagram showing the 414 human genes compared with DE genes in group 2 mice compared with group 1 (*n* = 285), TLS (*n* = 644), and lymph nodes genes (*n* = 1976). **F:** Venn diagram showing the 414 human genes compared with DE genes in group 3 mice compared with group 1 (*n* = 1436), TLS (*n* = 644), and lymph nodes genes (*n* = 1976). Group 1 (G1), young, antibody negative mice; group 2 (G2), 4 to 5 week anti-dsDNA antibody-positive mice; group 3 (G3), proteinuric mice.

The genes found in TLS and lymph nodes were further compared with the DE genes in group 2 and 3 mice compared with group 1 mice (Figure 5, A and B, and Supplemental Tables S5–S7). Most of the DE genes observed in groups 2 and 3 were immune system related (Figure 5, C–E). Of the 644 genes found DE in TLS, 133 were found in group 2 mice, and 201 were found in group 3 mice (Figure 6A and Supplemental Table S8). In addition, 52 and 186 lymph nodes genes were found in group 2 and 3 mice, respectively (Figure 6A and Supplemental Table S8). Thirty-four of these genes were found in the Gene Ontology term *hematopoietic or lymphoid organ development* (0048534). Table 5 lists the expression level in all comparisons. Protein-protein interaction networks analyses using STRING of the 130 common genes found in groups 2 and 3, and TLS had a strong T- and B-cell signature (Figure 6B). Comparing the total gene expression (all genes >0.5) in group 1 and 3 NZB/W mice and the DE genes in group 2 and 3 mice with published gene expression in kidneys of 8-week–old male C57BL/6J mice (*n* = 3)^29^ revealed 182 to 189 common genes (Supplemental Figure S1 and Supplemental Tables S9–S11). A large proportion of these were immune system–related genes.

**Table 5:** Group 2 and 3 Expression Level of Tertiary Lymphoid Structure Genes Found in the Gene Ontology Term *Hematopoietic or Lymphoid Organ Development*

Gene	Target	Description	Base Mean	Group 1 vs Group 2		Group 1 vs Group 3		Group 2 vs Group 3	
				logFC	PADJ	logFC	PADJ	logFC	PADJ
Apobec3	ENSMUSG00000009585	Apobec3, apolipoprotein B mRNA editing enzyme, catalytic polypeptide 3	232	NS		1.2	3.90 × 10^4^	NS	
*B2m*	ENSMUSG00000060802	B2m, β_2_-microglobulin	10,905	NS		1.5	1.08 × 10^12^	NS	
*Batf*	ENSMUSG00000034266	Batf, basic leucine zipper transcription factor, ATF-like	60	2.0	4.07 × 10^4^	2.5	1.09 × 10^7^	NS	
*Btk*	ENSMUSG00000031264	Btk, Bruton agammaglobulinemia tyrosine kinase	51	1.5	7.66 × 10^3^	2.4	5.83 × 10^8^	NS	
*Ccl19*	ENSMUSG00000071005	Ccl19, chemokine (C-C motif) ligand 19	75	2.5	1.07 × 10^4^	NS		−2.2	8.82 × 10^5^
*Ccr7*	ENSMUSG00000037944	Ccr7, chemokine (C-C motif) receptor 7	88	2.1	6.47 × 10^5^	NS		−1.2	1.14 × 10^2^
*Cd3d*	ENSMUSG00000032094	Cd3d, CD3 antigen, delta polypeptide	97	2.8	6.14 × 10^7^	1.8	4.37 × 10^4^	NS	
*Cd74*	ENSMUSG00000024610	Cd74, CD74 antigen (invariant polypeptide of major histocompatibility complex, class II antigen-associated)	8325	NS		2.2	4.39 × 10^24^	1.3	9.44 × 10^4^
*Cd79a*	ENSMUSG00000040592	Cd79b, CD79B antigen	121	NS		1.9	1.06 × 10^2^	NS	
*Cxcl13*	ENSMUSG00000023078	Cxcl13, chemokine (C-X-C motif) ligand 13	92	5.1	3.12 × 10^7^	5.8	1.50 × 10^10^	NS	
*Dock2*	ENSMUSG00000020143	Dock2, dedicator of cytokinesis 2	227	1.0	2.43 × 10^3^	1.2	9.48 × 10^6^	NS	
*H2ab1*	ENSMUSG00000073421	H2-Ab1, histocompatibility 2, class II antigen A, β1	837	NS		1.9	5.34 × 10^10^	1.1	6.67 × 10^4^
*H2-DMa*	ENSMUSG00000037649	H2-DMa, histocompatibility 2, class II, locus DMa	296	NS		1.7	1.38 × 10^8^	NS	
*Hcls1*	ENSMUSG00000022831	Hcls1, hematopoietic cell specific Lyn substrate 1	47	1.8	6.83 × 10^3^	2.8	5.80 × 10^8^	NS	
*Ikzf1*	ENSMUSG00000018654	Ikzf1, IKAROS family zinc finger 1	149	2.1	4.70 × 10^8^	2.4	7.62 × 10-^12^	NS	
*Irf1*	ENSMUSG00000018899	Irf1, interferon regulatory factor 1	1130	NS		1.2	1.20 × 10-0^9^	NS	
*Irf4*	ENSMUSG00000021356	Irf4, interferon regulatory factor 4	164	NS		1.9	5.55 × 10-0^3^	NS	
*Lck*	ENSMUSG00000000409	Lck, lymphocyte protein tyrosine kinase	66	2.5	5.56 × 10^5^	2.5	2.76 × 10-0^6^	NS	
*Lyn*	ENSMUSG00000042228	Lyn, Yamaguchi sarcoma viral (v-yes-1) oncogene homolog	819	NS		1.5	3.78 × 10-^11^	NS	
*Nckap1l*	ENSMUSG00000022488	Nckap1l, NCK associated protein 1 like	589	1.6	7.84 × 10^6^	2.3	3.68 × 10-^14^	NS	
*Pld4*	ENSMUSG00000052160	Pld4, phospholipase D family, member 4	417	1.5	1.80 × 10^3^	3.0	8.18 × 10-^15^	1.5	2.56 × 10^4^
*Plek*	ENSMUSG00000020120	Plek, pleckstrin	270	2.8	3.23 × 10^4^	3.7	2.10 × 10^8^	NS	
*Pou2f2*	ENSMUSG00000008496	Pou2f2, POU domain, class 2, transcription factor 2	534	2.5	2.63 × 10^5^	3.3	4.62 × 10^10^	NS	
*Ptpn22*	ENSMUSG00000027843	Ptpn22, protein tyrosine phosphatase, non-receptor type 22 (lymphoid)	256	NS		1.0	6.43 × 10^4^	NS	
*Ptprc*	ENSMUSG00000026395	Ptprc, protein tyrosine phosphatase, receptor type, C	97	1.8	1.53 × 10^2^	2.6	4.43 × 10^6^	NS	
*Siglecg*	ENSMUSG00000030468	Siglecg, sialic acid binding Ig-like lectin G	50	3.2	7.07 × 10^8^	2.5	9.63 × 10^6^	NS	
*Spn*	ENSMUSG00000051457	Spn, sialophorin	380	2.4	3.67 × 10^5^	3.1	6.25 × 10^10^	NS	
*Syk*	ENSMUSG00000021457	Syk, spleen tyrosine kinase	84	1.2	4.41 × 10^2^	1.9	1.05 × 10^5^	NS	
*Tcf7*	ENSMUSG00000000782	Tcf7, transcription factor 7, T cell specific	93	1.9	1.65 × 10^4^	1.6	3.18 × 10^4^	NS	
*Tnfrsf13b*	ENSMUSG00000010142	Tnfrsf13b, tumor necrosis factor receptor superfamily, member 13b	120	1.9	3.48 × 10^5^	2.7	1.73 × 10^11^	NS	
*Tyrobp*	ENSMUSG00000030579	Tyrobp, TYRO protein tyrosine kinase binding protein	330	NS		2.4	5.38 × 10^10^	1.6	6.41 × 10^5^
*Ubd*	ENSMUSG00000035186	Ubd, ubiquitin D	291	2.3	8.53 × 10^7^	2.4	7.29 × 10^9^	NS	
*Vav1*	ENSMUSG00000034116	Vav1, vav 1 oncogene	220	1.5	3.39 × 10^4^	2.3	7.42 × 10^11^	NS	
*Zfp36l2*	ENSMUSG00000045817	Zfp36l2, zinc finger protein 36, C3H type-like 2	182	NS		NS		1.5	4.05 × 10^7^

Data from *https://www.ensembl.org/index.html*, last accessed June 25, 2020.

logFC, log fold change; NS, not significant; PADJ, *P* adjusted.

To analyze whether the gene expression of TLS in kidneys was similar to gene expression of TLS observed in another mouse strain, the published data on TLS were compared within the chorus plexus of MRL lpr/lpr mice.^30^ The DE genes of chorus plexus TLS had 421 common genes with kidney TLS, 213 common genes with DE genes in group 2 mice, and 436 common genes compared with DE genes in group 3 mice (Figure 6C and Supplemental Table S12). The comparison found 126 common genes within all groups, and paired correlation plots on scaled log2-fold change of the genes revealed some of these (Figure 6D).

### Murine Expression Profiles Comparable to Human LN Gene Expression

**Figure 7:**
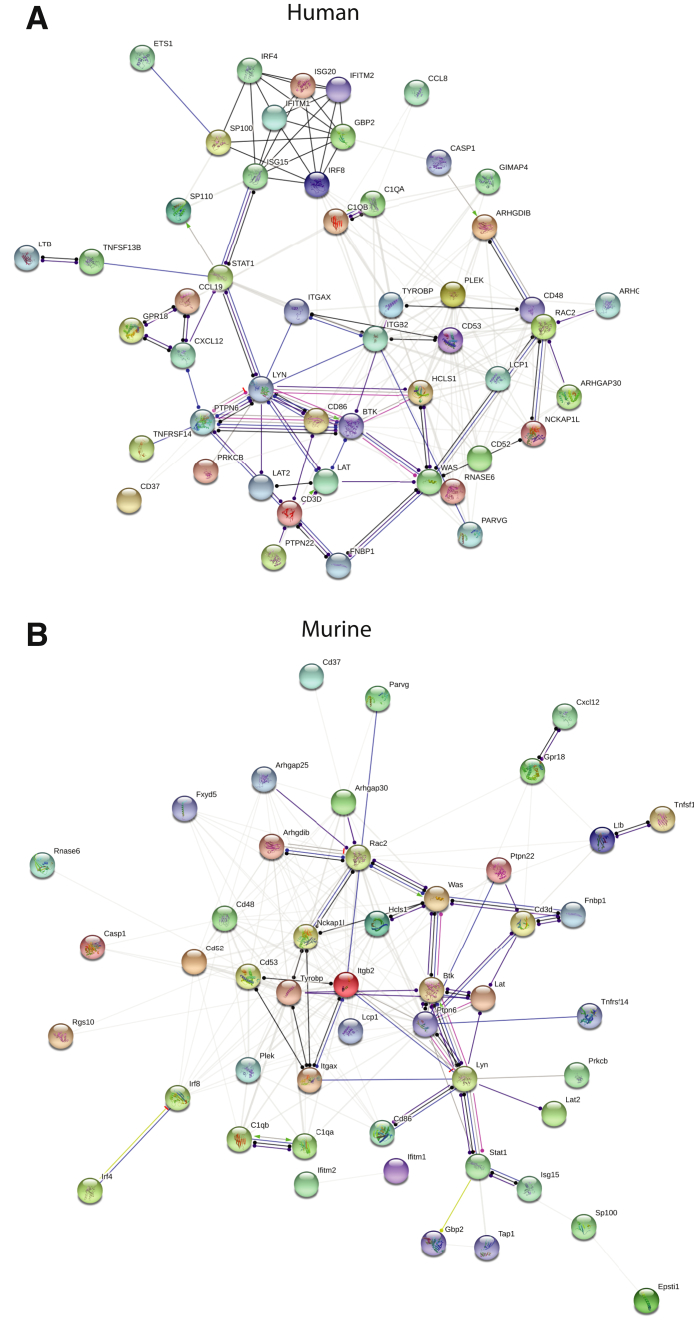
Search Tool for the Retrieval of Interacting Genes/Proteins interaction network of the 65 Tertiary lymphoid structure genes found in human lupus nephritis (LN) gene expression analyses. The genes were analyzed as human (**A**) or murine (**B**) genes.

**Figure 8:**
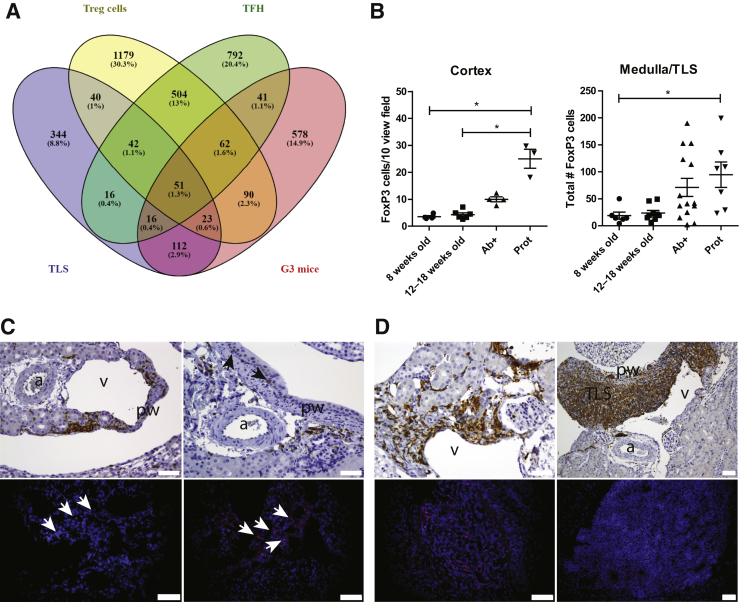
Infiltrating T-regulatory cells (Tregs) are detected within the pelvic wall and increase in both cortex and medulla during the development of lupus nephritis (LN). **A:** Venn diagram of tertiary lymphoid structure (TLS)–specific gene compared with genes found in human kidney infiltrating Tregs and follicular T-helper (TFH) cells and group 3 mice. The gene expression after single-cell sequencing cluster profiling of kidney infiltrating Tregs (*n* = 1990) and TFH cells (*n* = 1523) in LN were compared to the TLS-specific genes (*n* = 644) and the up-regulated genes in group 3 mice (*n* = 973). **B:** Forkhead box P3 (FoxP3)–positive cells were counted in 10 view fields at ×20 magnification within the cortex and the whole medulla/TLS of 4- to 8-week–old, 10- to 18-week–old, anti–double-stranded DNA antibody–positive (Ab^+^), and proteinuric (Prot) New Zealand black × New Zealand white (NZB/W) mice. FoxP3-positive cells increase in the cortex and medulla of mice in the Prot group. **C** and **D:** CD3-positive (**top rows**, brown cells and **black arrows**) and FoxP3 positive-cells (**bottom rows**, **white arrows** and pink cells) detected within the pelvic wall of young NZB/W mice (**C**) and Ab^+^ and Prot NZB/W mice (**D**). ∗*P* < 0.05. Scale bars: 50 μm (**C**, and **D**, **left column**); 20 μm (**D**, **right column**). a, artery; pw, pelvic wall; v, vein.

It was investigated whether the gene expression observed in murine kidneys, isolated TLS, and lymph nodes could be comparable to a recent published gene expression profile observed in human kidney biopsies from patients with SLE and LN^31^ and single-cell sequencing of immune cell profiles in similar patients with LN.^32^ Of the 414 human DE genes in patients with SLE compared with normal human kidney samples, 65 were found expressed in murine kidney TLS (Figure 6, E and F, and Supplemental Tables S13 and S14). Of these genes, 24 were found in group 2 mice, and 39 were found in group 3 mice. STRING interaction network analyses of the 65 genes were analyzed with the human and murine genes, revealing a similar interaction network, although with some differences (Figure 7). In addition to the genes found in TLS, 7 and 45 human genes were also found in group 2 and 3 mice, respectively (Figure 6, E and F). These genes were enriched in several biological processes (Table 6). Comparison of the gene expression in TLS and group 3 mice with the cell-specific gene clusters of T-regulatory cells (Tregs) and follicular T-helper (TFH) cells found in human patients with SLE revealed 133 Treg genes and 125 TFH genes in TLS (Figure 8A and Supplemental Table S15). In group 3 mice, 223 Treg genes and 160 TFH genes were found up-regulated (Figure 8A). The Tregs were quantified using immunohistochemistry and immunofluorescence-stained kidney sections. The number of Tregs increased in the cortex and medulla during the development of LN (Figure 8B). In the young mice, the Tregs were located within the pelvic wall close to the largest arteries and veins (Figure 8C), whereas in the older mice, the Tregs were increasingly found in the circulation within the glomeruli and tubuli interstitially. However, most Tregs were located within the TLS (Figure 8D). Analysis of all the single-cell profiles of immune cells found in human LN with the gene profile of murine TLS identified almost all genes differentially expressed in murine kidneys during LN development (data not shown).

**Table 6:** Genes Up-Regulated in Human LN and Murine LN Not Found in TLS or Lymph Nodes Were Enriched Following GO Terms

GO no.	Term Description	Observed Gene Count	Background Gene Count	False Discovery Rate	Matching Proteins in the Network (Labels)
0030198	Extracellular matrix organization	8	296	0.00061	ADAM19,COL1A1,COL1A2,COL3A1,COL6A3,LUM,PXDN,TNC
0030199	Collagen fibril organization	4	39	0.0014	COL1A1,COL1A2,COL3A1,LUM
0034097	Response to cytokine	12	1035	0.0014	ALDH1A2,ANXA1,AXL,CCR5,COL1A1,COL1A2,COL3A1,CXCL10,IFIT1,IFIT2,OAS2,XAF1
0071229	Cellular response to acid chemical	6	196	0.0020	ALDH1A2,COL1A1,COL1A2,COL3A1,PTGER4,TNC
0001501	Skeletal system development	8	457	0.0024	CDH11,COL1A1,COL1A2,COL3A1,FRZB,LUM,PTGER4,RUNX1
0009612	Response to mechanical stimulus	6	210	0.0024	COL1A1,COL3A1,CXCL10,PTGER4,SERPINE2,TNC
0042060	Wound healing	8	461	0.0024	ANXA1,AXL,COL1A1,COL1A2,COL3A1,MYOF,SERPINE2,TNC
0071396	Cellular response to lipid	8	486	0.0025	ALDH1A2,ANXA1,AXL,CCR5,COL1A1,CXCL10,PTGER4,TNC
0060337	Type I interferon signaling pathway	4	65	0.0029	IFIT1,IFIT2,OAS2,XAF1
1901701	Cellular response to oxygen-containing compound	10	896	0.0036	ALDH1A2,ANXA1,AXL,CCR5,COL1A1,COL1A2,COL3A1,CXCL10,PTGER4,TNC
0002376	Immune system process	16	2370	0.0045	ANXA1,AP1S2,AXL,CCR5,CFH,COL1A1,COL1A2,CXCL10,FGL2,IFIT1,IFIT2,OAS2,PTGER4,PXDN,RUNX1,XAF1
0009605	Response to external stimulus	14	1857	0.0046	ALDH1A2,ANXA1,AXL,CCR5,CMTM3,COL1A1,COL3A1,CXCL10,IFIT1,IFIT2,OAS2,PTGER4,SERPINE2,TNC
0071345	Cellular response to cytokine stimulus	10	953	0.0047	ANXA1,AXL,CCR5,COL1A1,COL1A2,CXCL10,IFIT1,IFIT2,OAS2,XAF1
0033273	Response to vitamin	4	87	0.0048	ALDH1A2,COL1A1,CXCL10,TNC
0071310	Cellular response to organic substance	15	2219	0.0067	ALDH1A2,ANXA1,AXL,CCR5,COL1A1,COL1A2,COL3A1,CXCL10,IFIT1,IFIT2,OAS2,PTGER4,RGMB,TNC,XAF1
0097350	Neutrophil clearance	2	5	0.0079	ANXA1,AXL
0007155	Cell adhesion	9	843	0.0080	AXL,CDH11,COL3A1,COL6A3,EMILIN2,PCDH18,RGMB,THBS2,TNC
0019221	Cytokine-mediated signaling pathway	8	655	0.0080	ANXA1,CCR5,COL1A2,CXCL10,IFIT1,IFIT2,OAS2,XAF1
0006955	Immune response	12	1560	0.0096	ANXA1,AXL,CCR5,CFH,CXCL10,FGL2,IFIT1,IFIT2,OAS2,PTGER4,PXDN,XAF1
0070848	Response to growth factor	7	507	0.0096	ANXA1,COL1A1,COL1A2,COL3A1,LUM,RGMB,TNC

Data from *http://geneontology.org/docs/go-enrichment-analysis*, last accessed June 25, 2020.

GO, Gene Ontology; LN, lupus nephritis; TLS, tertiary lymphoid structures.

## Discussion

Aggregates of lymphocytes resembling TLS have been described within the kidney of both human and murine LN.^15^^,^^33^, ^34^, ^35^ The development of fully functional and organized TLS has not previously been reported for the NZB/W mouse model. In this study, the formation of large TLS within the pelvic wall of the medulla and close to the arcuate arteries within the cortex of kidneys taken from lupus-prone NZB/W mice were found. The structures were organized in large, interconnected areas along the veins and arteries. It was found that the structures contain all cells necessary to function as an activation site or a site for regulating T and B cells.

The gene expression of TLS and the change in gene expression within the kidney during the development of LN resembled the gene expression in lymph nodes. Most of the up-regulated genes in the kidney were involved in the innate and adaptive immune system, whereas the down-regulated genes were mostly involved in metabolic processes. This finding agrees with previous studies on both human LN biopsies and other murine lupus models.^36^^,^^37^ Indeed, when the gene expression profile of TLS, lymph nodes, and group 2 and 3 mice were compared with the published gene expression profile of kidney biopsies from patients with SLE and LN, some of the genes were common.^31^ Among these genes, C1q, *Ptpn22*, and *Tnfsf13b* (BAFF) are involved in the development of LN.^38^, ^39^, ^40^

In addition to the strong gene signature of immunoglobulins, an early activation of T cells and natural killer cells in group 2 mice were identified. It has been previously reported that T cells are infiltrating the kidneys at an early stage.^5^^,^^22^ Many of the genes seen up-regulated in group 2 mice are involved in transcriptional and epigenetic regulation of TFH cells.^41^ TFH cells are essential for B-cell activation in germinal centers.^42^ Together with the expression of various immunoglobulin genes and the composition of B cells, FDCs, and TFH cells, this finding indicates that the TLS may have functional germinal centers that may be involved in the disease development. The local production of kidney-specific autoantibodies may transfer the disease from a silent nephritis to a clinical active disease. However, the gene expression profiles also indicate that the TLS may have a regulatory effect. FoxP3 Tregs were found in the cortex and medulla in addition to the TLS, increasing with disease progression. Tregs in SLE have been extensively studied, but they reveal a large heterogeneity and the results are contradictory.^43^ However, evidence indicates that an increased amount of Tregs are found in patients with SLE,^42^ especially within the kidneys of patients with LN.^44^^,^^45^ Interestingly, in recently published data, a unique thymus-derived Treg population was found to be increased in patients with SLE.^46^ Hanaoka et al^46^ also found infiltration of these Tregs within kidney biopsies from patients with active LN. The results from our comparison to the human single-cell gene expression data indicated a similar finding. In a study on kidney infiltrating T cells in MRL lpr mice, an exhausted phenotype was found,^47^ which could be caused by regulation (Treg) in TLS and thereby have an inhibitory effect on T cells. However, in a recently published article on single-cell sequencing of immune cells isolated from kidney biopsies of patients with LN, the authors did not find this exhausted phenotype.^32^ They found a subset of regulatory cells, particularly TFH regulatory cells, indicating that more studies on the role of TLS in kidney diseases are needed.

The role of macrophages, DCs, and B cells in the progression of LN has been extensively studied in humans and mice.^48^^,^^49^ In studies using DC depletion and DC plus B-cell Myd88 deletion, perivascular and peritubular infiltrates were decreased in DC-depleted and Myd88-deleted lupus-prone animals compared with sick control mice.^49^^,^^50^ Nevertheless, the fact that the peritubular infiltrates were diminished but still present indicates a role for cells other than DCs and B cells in the induction of TLS. This assumption was confirmed because B cells and DCs were not found in the early aggregates that consisted of mainly macrophages, T cells, and intrinsic kidney cells. The gene expression analyses found an increased expression of monocyte and macrophage-related genes to be up-regulated during the progression of the disease. A study by Schiffer et al^48^ found that macrophages are one of the first immune cells to be activated and increased in the development of LN. They also found that the effect of interferon-α not only activates the innate immune system but that the anti-dsDNA antibody induction and clinical SLE driven by interferon-α are dependent on CD4^+^ T cells.^51^ The activation and infiltration of T cells within the kidney and the formation of TLS need to be further studied to determine whether TLS have an active role in driving the disease from a silent to a clinical active disease or whether TLS may have an inhibitory effect on the disease progression.

The current study results are in line with and extend previous results because structures that resembled TLS in the cortex of human kidney biopsies taken from patients with SLE and LN were associated with tubular basement membrane immune complexes.^12^ Clark et al^12^ described three patterns of tubulointerstitial infiltrates in the kidneys of patients with SLE, starting from diffuse scattered, lymphocytic infiltration and continuing through T/B aggregates to germinal centers (characterized by the spatial cellular organization and FDCs). The germinal centers were found for only 6% of the biopsies analyzed. In a retrospective study on kidney biopsies from patients with IgA nephropathies, a strong association with TLS formation and the severity of disease was found.^11^ Patients with high-grade TLS exhibited a higher percentage of mesangial hypercellularity and crescents as well as severe interstitial and arterial lesions.^11^ In the current study's findings in kidneys of lupus-prone mice, the same patterns could be observed, often within the same kidney, and sometimes interconnected to larger structures in the hilum. Given that the human kidney biopsies are mainly taken from the cortical region of the kidneys, it is interesting that huge aggregates within the pelvic area of the kidney can be observed. We hypothesize that the kidney of patients with SLE may develop the same large structures because we observed similar patterns of immune cells within the cortex of murine kidneys as within human LN biopsies. However, the discovery of large TLS in human kidneys is unlikely with current methods because the detection of these structures is dependent on kidney biopsies. Developing new *in vivo* imaging methods, such as positron emission tomography/magnetic resonance imaging, to detect these structures at an early phase may lead to early diagnosis and better treatment strategies.

## Supplemental Data

Supplemental Figure S1 Venn diagram of total kidney gene expression of New Zealand black × New Zealand white (NZB/W) group 1 (**A**), group 2 (**B**), and group 3 (**C**) mice compared with C57BL/6J and differentially expressed (DE) genes in group 2 and 3 mice.

## References

[1] Rekvig O.P., Thiyagarajan D., Pedersen H.L., Horvei K.D., Seredkina N. (2016). Future perspectives on pathogenesis of lupus nephritis: facts, problems, and potential causal therapy modalities. Am J Pathol.

[2] Seredkina N., van der Vlag J., Berden J., Mortensen E., Rekvig O.P. (2013). Lupus nephritis - enigmas, conflicting models and an emerging concept. Mol Med.

[3] Fenton K., Fismen S., Hedberg A., Seredkina N., Fenton C., Mortensen E.S., Rekvig O.P. (2009). Anti-dsDNA antibodies promote initiation, and acquired loss of renal Dnase1 promotes progression of lupus nephritis in autoimmune (NZB×NZW)F1 mice. PLoS One.

[4] Kalaaji M., Fenton K.A., Mortensen E.S., Olsen R., Sturfelt G., Alm P., Rekvig O.P. (2007). Glomerular apoptotic nucleosomes are central target structures for nephritogenic antibodies in human SLE nephritis. Kidney Int.

[5] Kanapathippillai P., Hedberg A., Fenton C.G., Fenton K.A. (2013). Nucleosomes contribute to increase mesangial cell chemokine expression during the development of lupus nephritis. Cytokine.

[6] Yung S., Cheung K.F., Zhang Q., Chan T.M. (2013). Mediators of inflammation and their effect on resident renal cells: implications in lupus nephritis. Clin Dev Immunol.

[7] Maria N.I., Davidson A. (2017). Renal macrophages and dendritic cells in SLE nephritis. Curr Rheumatol Rep.

[8] Tsokos G.C., Lo M.S., Costa R.P., Sullivan K.E. (2016). New insights into the immunopathogenesis of systemic lupus erythematosus. Nat Rev Rheumatol.

[9] Aloisi F., Pujol-Borrell R. (2006). Lymphoid neogenesis in chronic inflammatory diseases. Nat Rev Immunol.

[10] van de Pavert S.A., Mebius R.E. (2010). New insights into the development of lymphoid tissues. Nat Rev Immunol.

[11] Pei G., Zeng R., Han M., Liao P., Zhou X., Li Y., Zhang Y., Liu P., Zhang C., Liu X., Yao Y., Xu G. (2014). Renal interstitial infiltration and tertiary lymphoid organ neogenesis in IgA nephropathy. Clin J Am Soc Nephrol.

[12] Chang A., Henderson S.G., Brandt D., Liu N., Guttikonda R., Hsieh C., Kaverina N., Utset T.O., Meehan S.M., Quigg R.J., Meffre E., Clark M.R. (2011). In situ B cell-mediated immune responses and tubulointerstitial inflammation in human lupus nephritis. J Immunol.

[13] He N., Chen W.L., Long K.X., Zhang X., Dong G.F. (2016). Association of serum CXCL13 with intrarenal ectopic lymphoid tissue formation in lupus nephritis. J Immunol Res.

[14] Kelly F.M., Reddy R.N., Roberts B.R., Gangappa S., Williams I.R., Gooch J.L. (2009). TGF-beta upregulation drives tertiary lymphoid organ formation and kidney dysfunction in calcineurin A-alpha heterozygous mice. Am J Physiol Renal Physiol.

[15] Kang S., Fedoriw Y., Brenneman E.K., Truong Y.K., Kikly K., Vilen B.J. (2017). BAFF induces tertiary lymphoid structures and positions T cells within the glomeruli during lupus nephritis. J Immunol.

[16] Huang Y., Caputo C.R., Noordmans G.A., Yazdani S., Monteiro L.H., van den Born J., van Goor H., Heeringa P., Korstanje R., Hillebrands J.L. (2014). Identification of novel genes associated with renal tertiary lymphoid organ formation in aging mice. PLoS One.

[17] Sato Y., Mii A., Hamazaki Y., Fujita H., Nakata H., Masuda K., Nishiyama S., Shibuya S., Haga H., Ogawa O., Shimizu A., Narumiya S., Kaisho T., Arita M., Yanagisawa M., Miyasaka M., Sharma K., Minato N., Kawamoto H., Yanagita M. (2016). Heterogeneous fibroblasts underlie age-dependent tertiary lymphoid tissues in the kidney. JCI Insight.

[18] Bethunaickan R., Berthier C.C., Zhang W., Kretzler M., Davidson A. (2013). Comparative transcriptional profiling of 3 murine models of SLE nephritis reveals both unique and shared regulatory networks. PLoS One.

[19] Berthier C.C., Bethunaickan R., Gonzalez-Rivera T., Nair V., Ramanujam M., Zhang W., Bottinger E.P., Segerer S., Lindenmeyer M., Cohen C.D., Davidson A., Kretzler M. (2012). Cross-species transcriptional network analysis defines shared inflammatory responses in murine and human lupus nephritis. J Immunol.

[20] Berthier C.C., Kretzler M., Davidson A. (2017). A systems approach to renal inflammation in SLE. Clin Immunol.

[21] Berthier C.C., Kretzler M., Davidson A. (2012). From the large scale expression analysis of lupus nephritis to targeted molecular medicine. J Data Mining Genomics Proteomics.

[22] Dorraji S.E., Hovd A.K., Kanapathippillai P., Bakland G., Eilertsen G.O., Figenschau S.L., Fenton K.A. (2018). Mesenchymal stem cells and T cells in the formation of tertiary lymphoid structures in lupus nephritis. Sci Rep.

[23] Kalaaji M., Sturfelt G., Mjelle J.E., Nossent H., Rekvig O.P. (2006). Critical comparative analyses of anti-alpha-actinin and glomerulus-bound antibodies in human and murine lupus nephritis. Arthritis Rheum.

[24] Rekvig O.P., Moens U., Sundsfjord A., Bredholt G., Osei A., Haaheim H., Traavik T., Arnesen E., Haga H.J. (1997). Experimental expression in mice and spontaneous expression in human SLE of polyomavirus T-antigen: a molecular basis for induction of antibodies to DNA and eukaryotic transcription factors. J Clin Invest.

[25] Tillman D.M., Jou N.T., Hill R.J., Marion T.N. (1992). Both IgM and IgG anti-DNA antibodies are the products of clonally selective B cell stimulation in (NZB × NZW)F1 mice. J Exp Med.

[26] Aringer M., Costenbader K., Daikh D., Brinks R., Mosca M., Ramsey-Goldman R. (2019). 2019 European League Against Rheumatism/American College of Rheumatology classification criteria for systemic lupus erythematosus. Arthritis Rheumatol.

[27] Robinson M.D., Oshlack A. (2010). A scaling normalization method for differential expression analysis of RNA-seq data. Genome Biol.

[28] Robinson M.D., McCarthy D.J., Smyth G.K. (2010). edgeR: a Bioconductor package for differential expression analysis of digital gene expression data. Bioinformatics.

[29] Clark J.Z., Chen L., Chou C.L., Jung H.J., Lee J.W., Knepper M.A. (2019). Representation and relative abundance of cell-type selective markers in whole-kidney RNA-Seq data. Kidney Int.

[30] Stock A.D., Der E., Gelb S., Huang M., Weidenheim K., Ben-Zvi A., Putterman C. (2019). Tertiary lymphoid structures in the choroid plexus in neuropsychiatric lupus. JCI Insight.

[31] Pamfil C., Makowska Z., De Groof A., Tilman G., Babaei S., Galant C., Montigny P., Demoulin N., Jadoul M., Aydin S., Lesche R., McDonald F., Houssiau F.A., Lauwerys B.R. (2018). Intrarenal activation of adaptive immune effectors is associated with tubular damage and impaired renal function in lupus nephritis. Ann Rheum Dis.

[32] Arazi A., Rao D.A., Berthier C.C., Davidson A., Liu Y., Hoover P.J. (2019). Accelerating medicines partnership in SLEn: the immune cell landscape in kidneys of patients with lupus nephritis. Nat Immunol.

[33] Adalid-Peralta L., Mathian A., Tran T., Delbos L., Durand-Gasselin I., Berrebi D., Peuchmaur M., Couderc J., Emilie D., Koutouzov S. (2008). Leukocytes and the kidney contribute to interstitial inflammation in lupus nephritis. Kidney Int.

[34] Davidson A., Aranow C. (2010). Lupus nephritis: lessons from murine models. Nat Rev Rheumatol.

[35] Shen Y., Sun C.Y., Wu F.X., Chen Y., Dai M., Yan Y.C., Yang C.D. (2012). Association of intrarenal B-cell infiltrates with clinical outcome in lupus nephritis: a study of 192 cases. Clin Dev Immunol.

[36] Rai G., Rai R., Saeidian A.H., Rai M. (2016). Microarray to deep sequencing: transcriptome and miRNA profiling to elucidate molecular pathways in systemic lupus erythematosus. Immunol Res.

[37] Gardet A., Chou W.C., Reynolds T.L., Velez D.B., Fu K., Czerkowicz J.M., Bajko J., Ranger A.M., Allaire N., Kerns H.M., Ryan S., Legault H.M., Dunstan R.W., Lafyatis R., Lukashev M., Viney J.L., Browning J.L., Rabah D. (2016). Pristane-accelerated autoimmune disease in (SWR X NZB) F1 mice leads to prominent tubulointerstitial inflammation and human lupus nephritis-like fibrosis. PLoS One.

[38] O'Flynn J., Flierman R., van der Pol P., Rops A., Satchell S.C., Mathieson P.W., van K.C., van der Vlag J., Berden J.H., Daha M.R. (2011). Nucleosomes and C1q bound to glomerular endothelial cells serve as targets for autoantibodies and determine complement activation. Mol Immunol.

[39] Mohan C., Putterman C. (2015). Genetics and pathogenesis of systemic lupus erythematosus and lupus nephritis. Nat Rev Nephrol.

[40] Collison J. (2017). Lupus nephritis: novel role for BAFF in tertiary lymphoid neogenesis. Nat Rev Rheumatol.

[41] Qiu H., Wu H., Chan V., Lau C.S., Lu Q. (2017). Transcriptional and epigenetic regulation of follicular T-helper cells and their role in autoimmunity. Autoimmunity.

[42] Linterman M.A., Rigby R.J., Wong R.K., Yu D., Brink R., Cannons J.L., Schwartzberg P.L., Cook M.C., Walters G.D., Vinuesa C.G. (2009). Follicular helper T cells are required for systemic autoimmunity. J Exp Med.

[43] Li W., Deng C., Yang H., Wang G. (2019). The regulatory T cell in active systemic lupus erythematosus patients: a systemic review and meta-analysis. Front Immunol.

[44] Ferreira R.C., Castro Dopico X., Oliveira J.J., Rainbow D.B., Yang J.H., Trzupek D., Todd S.A., McNeill M., Steri M., Orru V., Fiorillo E., Crouch D.J.M., Pekalski M.L., Cucca F., Tree T.I., Vyse T.J., Wicker L.S., Todd J.A. (2019). Chronic immune activation in systemic lupus erythematosus and the autoimmune PTPN22 Trp(620) risk allele drive the expansion of FOXP3(+) regulatory T cells and PD-1 expression. Front Immunol.

[45] Shakweer M.M., Behairy M., Elhefnawy N.G., Elsaid T.W. (2016). Value of Foxp3 expressing T-regulatory cells in renal tissue in lupus nephritis; an immunohistochemical study. J Nephropathol.

[46] Hanaoka H., Nishimoto T., Okazaki Y., Takeuchi T., Kuwana M. (2020). A unique thymus-derived regulatory T cell subset associated with systemic lupus erythematosus. Arthritis Res Ther.

[47] Tilstra J.S., Avery L., Menk A.V., Gordon R.A., Smita S., Kane L.P., Chikina M., Delgoffe G.M., Shlomchik M.J. (2018). Kidney-infiltrating T cells in murine lupus nephritis are metabolically and functionally exhausted. J Clin Invest.

[48] Schiffer L., Bethunaickan R., Ramanujam M., Huang W., Schiffer M., Tao H., Madaio M.P., Bottinger E.P., Davidson A. (2008). Activated renal macrophages are markers of disease onset and disease remission in lupus nephritis. J Immunol.

[49] Teichmann L.L., Ols M.L., Kashgarian M., Reizis B., Kaplan D.H., Shlomchik M.J. (2010). Dendritic cells in lupus are not required for activation of T and B cells but promote their expansion, resulting in tissue damage. Immunity.

[50] Teichmann L.L., Schenten D., Medzhitov R., Kashgarian M., Shlomchik M.J. (2013). Signals via the adaptor MyD88 in B cells and DCs make distinct and synergistic contributions to immune activation and tissue damage in lupus. Immunity.

[51] Liu Z., Bethunaickan R., Huang W., Lodhi U., Solano I., Madaio M.P., Davidson A. (2011). Interferon-alpha accelerates murine systemic lupus erythematosus in a T cell-dependent manner. Arthritis Rheum.

