# Supplementary figures and images for: Kidney Tertiary Lymphoid Structures in Lupus Nephritis Develop into Large Interconnected Networks and Resemble Lymph Nodes in Gene Signature
Source: Am J Pathol. 2020 Aug 17;190(11):2203–25. doi: 10.1016/j.ajpath.2020.07.015 (PMC12178333; doi:10.1016/j.ajpath.2020.07.015)

A

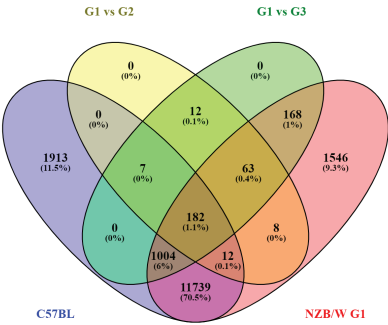

B

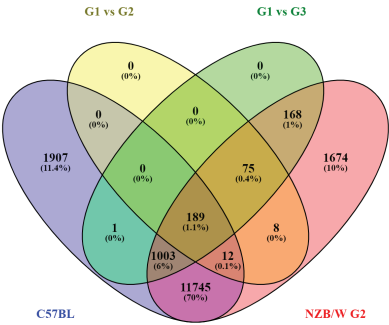

C

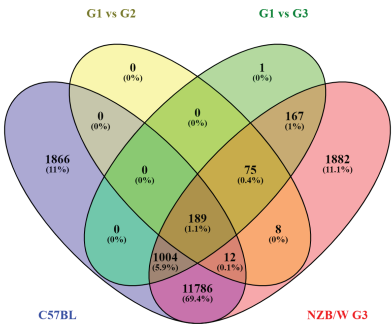

Supplement: Supplemental Figure S1 — Venn diagram of total kidney gene expression of New Zealand black × New Zealand white (NZB/W) group 1 (A), group 2 (B), and group 3 (C) mice compared with C57BL/6J and differentially expressed (DE) genes in group 2 and 3 mice. [file mmc1.pdf]
